# Progress of the Egyptian National Newborn Hearing Screening (ENHS) Program over a Four-Year Period

**DOI:** 10.3390/ijns11040108

**Published:** 2025-11-18

**Authors:** Eman Abdelbadei, Ahmed Mustafa, Abir Omara, Wafaa Shehata-Dieler, Mohamed Hassany

**Affiliations:** 1 Hearing and Speech Institute, Cairo 12511, Egypt; eman_ghorab2003@yahoo.com (E.A.); am.mustafa@windowslive.com (A.M.); abir_omara@yahoo.com (A.O.); 2 Presidential Initiative, Cairo 11863, Egypt; mohamed.hassany@mohp.gov.eg; 3 Comprehensive Hearing Center (CHC), Department of Otorhinolaryngology-Head & Neck Surgery, Wuerzburg University Hospitals, 97080 Wuerzburg, Germany; 4 Egyptian Ministry of Health and Population, Cairo 11516, Egypt

**Keywords:** universal neonatal hearing screening, quality measures, otoacoustic emissions

## Abstract

Universal newborn hearing screening (UNHS) has become widely adopted worldwide as a standard of care for the early detection of congenital hearing loss. The Egyptian UNHS program started as a presidential initiative by the Ministry of Health in November 2019. The program was initiated in 1346 primary health care units (PHCUs) located throughout the 26 governorates. A retrospective study was conducted to assess the performance of the Egyptian Program during the period from November 2019 to July 2023. Quality measures recommended by the Joint Committee on Infant Hearing including coverage rate, rate of referral to a second screening, follow up rate of attendance of second screening, referral for diagnosis rate, and follow up rate of attendance of diagnostic assessment, were analyzed. Over a period of 3 years and 9 months, more than five and half million infants underwent a first screening. The coverage rate was initially 39% and increased to reach 82% in 2023. The rate of referral to a second screen was 7.2% in 2019 and reached 5.2% in 2023. The follow-up rate of attendance of a second screening improved throughout the study period, from 75.5% to 92.1% but did not reach the benchmark of 95%. The rate of referrals for diagnosis was less than 1.7% and rate of attendance of a diagnostic assessment was initially 20% and improved to more than 65% in 2023. The very low rate of attendance of diagnostic assessment in 2020 and 2021 was attributed to the effects of the COVID pandemic.

## 1. Introduction

Congenital hearing loss is one of the most common disabilities in neonates, with a prevalence of 1 to 3 per 1000 live newborns [1]. Recent estimates by the World Health Organization (WHO) have indicated a growing absolute number and prevalence of people with disabling hearing loss [2,3]. According to Neumann et al. [4], 34 million children are estimated to have disabling hearing loss, most of them living in South Asia, Asia Pacific, and Sub-Saharan Africa [3,5,6]. These children are in danger of impaired language, social, emotional, and academic development [7,8,9].

Early hearing detection and intervention is a concept that started in the USA in the 1990s and constitutes a neonatal hearing screening prior to hospital discharge. The Joint Committee on Infant Hearing (JCIH) [10] recommends that all infants be screened for hearing loss at latest age of 1 month, diagnosed at age 3 months, and receive intervention at 6 months at the latest.

Universal newborn hearing screening (UNHS) allows for early management, which minimizes auditory deprivation while maximizing the auditory stimulation during the peak period of neural growth. This subsequently minimizes the deleterious impact of hearing loss (HL) on the language, cognitive, and psychosocial development of children diagnosed with congenital hearing loss [2].

Over the past 25 years, several countries have implemented UNHS programs. However, numerous UNHS programs faced significant difficulties at initiation [11,12,13].

The Egyptian Newborn Hearing Screening (ENHS) program started as a presidential initiative by the Ministry of Health in November 2019. The program follows the JCIH guidelines to identify newborns with congenital hearing loss by the age of 3 months and to initiate appropriate intervention by 6 months [10]. The guidelines also promote continuous quality assessment to track the program’s performance. Quality indicators from the guidelines include the coverage rate, rate of referral to a second screening, rate of attendance of second screenings, referral for diagnosis rate, and rate of attendance of diagnosis appointments.

This study aimed to assess the performance of the Egyptian program in the primary health care units (PHCUs) over a period of almost four years and to determine whether the program meets the recommended international benchmarks.

## 2. Methods

### 2.1. Preparation for ENHS

A selected committee was assigned by the Egyptian government and Ministry of Health. The duties of this committee included the initiation of the UNHS program, the development of a protocol for ENHS, providing the equipment needed for screening, training the screening personnel, and monitoring and following the program. A centralized screening center responsible for ENHS was established at the Hearing and Speech Institute in Cairo and a data management system to connect PHCUs with the NHS center and with the referral hospitals was developed by the Ministry of Health.

### 2.2. Screening Methods

Egyptian’s national hearing screening program implements a two-stage-screening protocol. The first stage is performed together with thyroid screening at the age of one week; if a second screen is needed, it is performed one to two weeks later at the age of two to three weeks.

Babies are screened in primary health care units (PHCUs), which are distributed throughout the country. The screening method is the Otoacoustic Emissions (OAE) test. During preliminary testing in 20 PHCUs, TEOAE and DPOAE were compared (pilot study), and TEOAE testing showed a very high “REFER” rate (14–23%). This was mainly attributed to the noisy environment in PHCUs. This was the case despite assigning a separate, relatively quiet room for hearing screening. The DPOAE measurements were less vulnerable to ambient noise and showed much lower “REFER” rates of 7–10%. Accordingly, it was decided to implement DPOAE testing as the screening method.

Two OAE screening devices were used for the OAE measurements (ERO•SCAN^®^ by MAICO Diagnostics GmbH, Berlin, Germany, and OtoRead™ by Interacoustics A/S, Middelfart, Denmark). Both devices automatically measure the otoacoustic emissions and if the response amplitude rises above its preset noise-floor thresholds across the required frequencies, it displays “PASS”; otherwise, a “REFER” result is provided. All screening devices received regular technical checks and were serviced at regular intervals.

The first screening is performed one week after birth in combination with the long-established thyroid screening in PHCUs distributed throughout all 26 governorates. The screening test is performed once. If the test is interrupted by noise or baby movement, it is repeated. A completed test with a “PASS” or “REFER” result is not repeated. If the first screening result is “REFER”, a follow-up screening (second screening) is performed at the same PHCU one to two weeks later. Parents receive written information on the procedures and timing of the thyroid and hearing screening directly after birth.

The testing is performed by trained nurses in a quiet room in the PHCU. The personnel conducting the screening are required to attend an initial intense training course on testing and data management. This is followed by additional training courses at regular intervals to ensure the retention of knowledge and skill of the screeners. Training courses, supervision, and continuous support for the PHCU personnel are provided by the audiologists from the NHS center at the Hearing and Speech Institute in Cairo.

**Figure 1:**
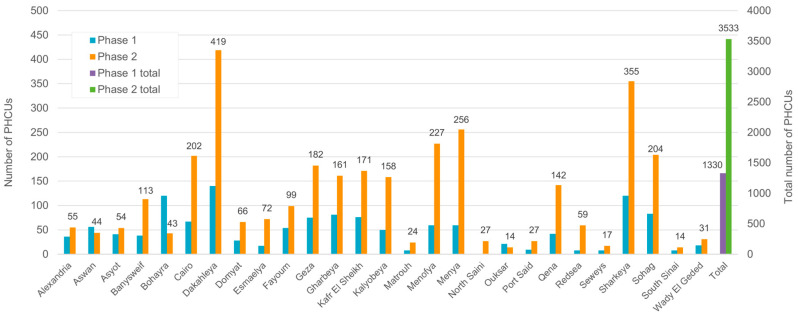
Number of PHCUs in different governorates performing neonatal hearing screening in the first and second phases and total number of PHCUs.

In the first phase of implementing the program (until the end of 2020), screening was performed in 1364 PHCUs. As of 2021, the number of screening centers was increased to 3500 (second phase). The PHCUs selected in the first and second phase were distributed throughout all 26 Egyptian governorates. The number of PHCUs in the different governorates performing the screening in the first and second phases, as well as the total number of PHCUs, is displayed in Figure 1.

### 2.3. Diagnosis

Infants who fail the second screening are referred to one of the specialized diagnostic audiology departments located in 31 hospitals distributed throughout the country. The PHCU staff direct the parents to the nearest hospital depending on their place of residence.

In the audiology departments, an otoscopic ear inspection is performed to exclude the presence of wax and evaluate the tympanic membrane as well as the middle ear condition. This is followed by audiological assessment using OAE, high-frequency tympanometry, and auditory brainstem response (ABR) measurements. Infants with abnormal click ABR thresholds were further evaluated using frequency-specific ABR and auditory steady-state response (ASSR) testing. Based on the results of these tests, the type and extent of hearing loss is determined and finally, infants with hearing loss are referred for intervention. The intervention includes hearing aids and/or medical treatment for middle ear ventilation disorders consisting of physiological saline solution in form of inhalation and nasal drops for younger infants. Infants older than 6 months receive, together with saline solution, decongestant nasal drops with age-appropriate concentration. Cochlear implants are provided to children with severe-to-profound hearing loss who, after a trial period, did not benefit from hearing aids.

### 2.4. Data Management

The centralized screening center located in the Hearing and Speech Institute in Cairo is exclusively responsible for NHS and supervises the screening units all over the country. It receives daily screening results and is responsible for tracking neonates who were referred for a second screening. Furthermore, infants referred for a diagnostic evaluation are tracked until a final diagnosis of the type and degree of hearing loss is reached and results of the applied intervention are obtained. An Egyptian national data management system was designed and implemented by the Ministry of Health and connected to the server of birth data; it is located in the NHS center at the Hearing and Speech Institute in Cairo. This system contains data of the first screening stage and second screening stage, the number of infants referred for diagnosis, and follow-up data for infants referred to specialized audiology departments. Follow-up is ensured through several steps beginning with written information given to the parents at the PHCUs, followed by phone calls and house visits by personnel from the tracking center:

1. Newborns who fail the first screening receive an appointment for a second screening one to two weeks later at the same PHCU. If the family do not show up after one month, they are contacted by phone by PHCU personnel two times in two different weeks.
2. If they still do not respond, they are reported to the screening center of the Ministry of Health, which contacts them by phone or visits them at home.
3. If the parents had the second screening performed in a private hospital, they are still urged to visit the PHCUs or send the results for documentation.

The parents of infants who fail the second screening are given the address of the audiology department in hospitals close to their place of residence. The data of the children is forwarded to the corresponding hospitals, which in turn contact the parents and offer them appointments within 3 to 6 weeks. If the parents do not show up before their child is 6 months of age, they are contacted again by phone and the personnel from the tracking center will eventually conduct a house visit.

The database stores the registration name and national ID specified for every PHCU to protect the patients’ information and privacy and the program’s confidentiality.

### 2.5. The Following Quality Measures Recommended by the JCIH [10] Were Analyzed

1. Coverage rate: Number of infants screened/total live births (≥95%).
2. Initial referral rate: Number of infants that failed the first screening/number of screened infants (≤4%).
3. Follow-up rate: Number of infants attending a second screening/number of infants that failed the first screening (≥95%).
4. Rate of referral for diagnosis and follow up attendance rate of diagnostic assessment.

For all four benchmarks, we included the terms used by the JCIH recommendations and by Neuman et al. 2020 [4] to allow for easier international comparisons.

## 3. Results

This study analyzed the performance of the Egyptian UNHS program conducted in the PHCUs over 3 years and 9 months.

### 3.1. Coverage Rate (Coverage of Screening)

**Figure 2:**
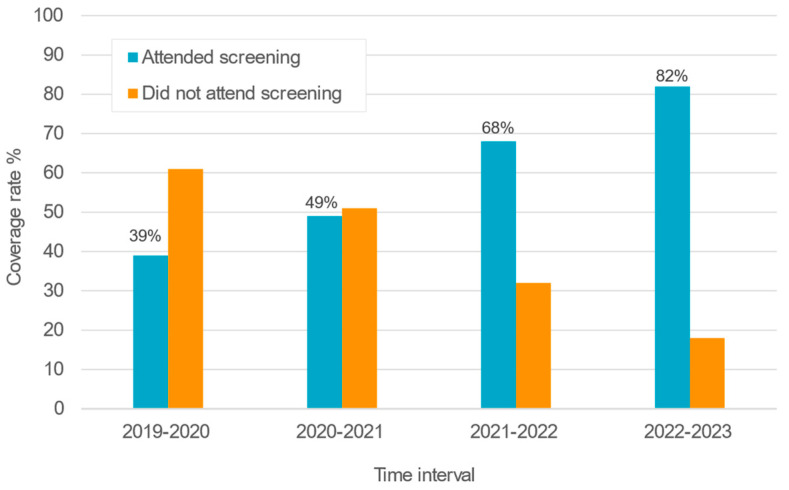
Percentage of newborns that attended the first screening (coverage rate).

**Figure 3:**
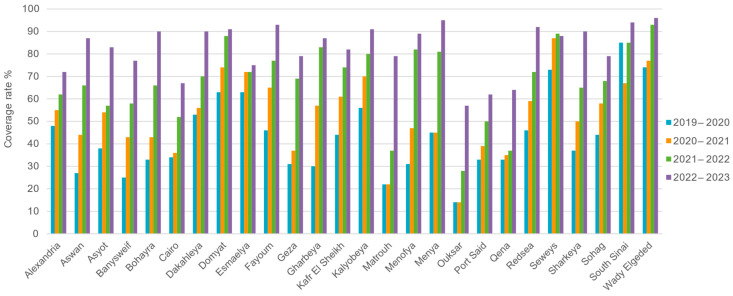
Percentage of newborns that attended the first screening (coverage rate) in the 26 governorates through the evaluation period.

Over the 3 years and 9 months, 5,118,402 infants attended the initial screening. The coverage rate refers to the percentage of newborns who attended the initial screening out of the total number of babies born during the same time. Figure 2 and Figure 3 shown that the coverage rate was only 39% at the beginning but increased over time and reached 82% in 2023. Figure 3 shows the increase in number of screened infants and coverage rate over time in the different governorates. The percentage of infants that underwent screening varied between the different governorates. However, by the end of the evaluation period, the coverage rates of the different governorates were comparable.

One unexpected factor affecting the coverage rate was the COVID pandemic. The program was initiated in the PHCUs in the first half of November 2019. During a period of 3.5 months until the beginning of March 2020, the number of newborns examined was 432 562 (64.3% of total births), while in the following 4 months, the number of screenings decreased to 240 330 (35.7% of total births).

The initial coverage rate varied from 14% to 85% across the different regions and reached the benchmark of ≥95% in two regions (Menya and Wady El Geded) (Figure 3).

### 3.2. Initial Referral Rate (Fail Rate After First Screening)

The first screening in the Egyptian program is performed one week after delivery in the PHCUs. The test is performed once. If the test is interrupted by noise or baby movement, it is repeated. A completed test with a “PASS” or “REFER” result is not repeated. The initial referral rate is the percentage of newborns who failed the initial test in either one or both ears. According to the JCIH benchmark, this value must be below 4%.

Table 1 shows a declining trend for the initial referral rate. The rate was 7.2% during the initial interval from November 2019 to July 2020. The referral rate, however, decreased during the following years and dropped to around 5% during the following time intervals until July 2023. This improvement was observed despite the total number of babies screened increasing from 749,872 during the first time interval to 1,846,114 babies during the last time interval.

### 3.3. Follow-Up Rate and Lost-to-Follow-Up Rate

In Egypt, the percentage newborns that attended the second screening at the beginning of the program was relatively low, but it improved continuously throughout the study period, ranging from 75.5% in the first year to 92.1% in the last year (Table 2). However, it has not reached the benchmark of 95%.

**Figure 4:**
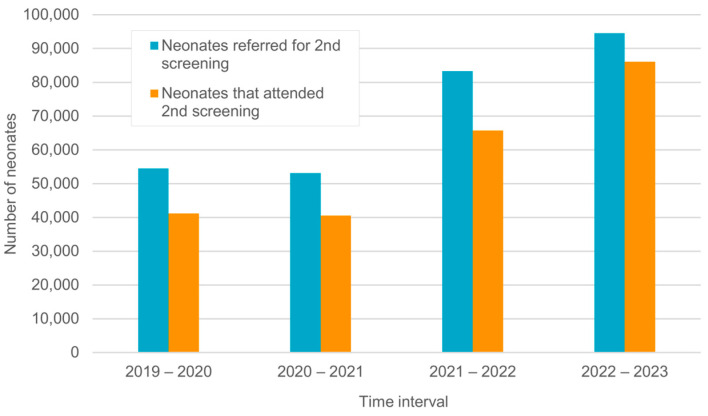
Progress in the number of neonates who attended a second 2nd screening.

Throughout the rollout of the screening program, the number of neonates requiring a second screening increased from 54,510 to 94,526 as the coverage rate increased. Figure 4 illustrates the decreasing gap between neonates referred to second screening and those attending a second screening.

### 3.4. Rate of Referral for Diagnosis, Follow Up Attendance Rate of Diagnostic Assessment and Lost-to-Follow-Up Rate for Diagnosis

**Figure 5:**
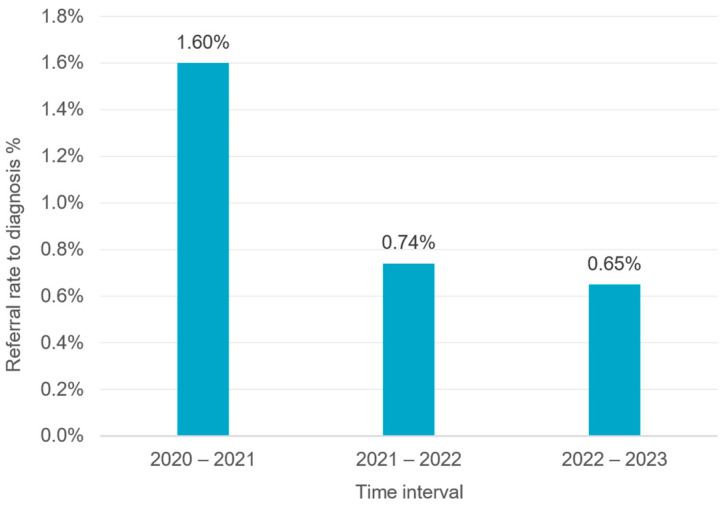
Percentage of infants referred for audiological assessment in hospitals.

**Figure 6:**
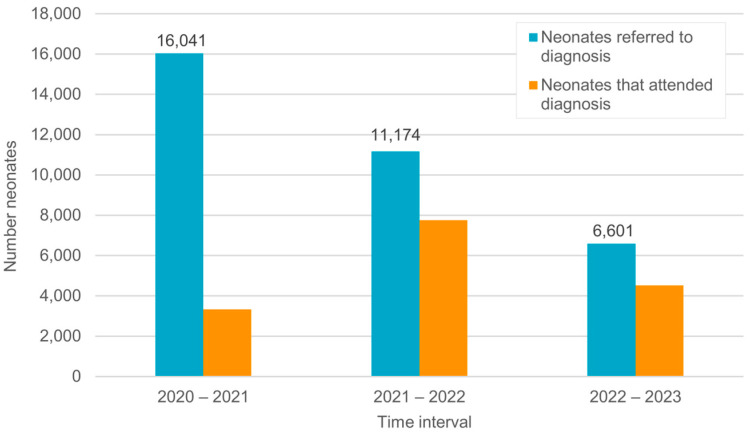
Number of infants referred for and number that attended audiological assessments in hospitals.

Infants who failed the second screening were referred to one of the 31 highly equipped Ministry of Health specialized diagnostic audiology departments in hospitals distributed throughout the country. During the evaluation period, the rate of referral for diagnosis in the Egyptian program was relatively low. Less than 1.7% of the infants who underwent a second screening were referred for diagnosis. This number continuously decreased to less than 1% during the following time intervals (Figure 5), i.e., more than 98.3–99% of infants who underwent a second screening received a “PASS” result and no further evaluation was necessary. On the other hand, at the beginning of the program, only a small portion of infants who did not achieve a “PASS” in the second screening and were referred for audiological assessment attended their diagnosis appointment. From the 16,041 infants referred for diagnosis, only 3326 (20.73%) attended the diagnosis appointment during the first time interval (2020–2021) (Figure 6). As shown in Figure 6, the number of infants receiving diagnostic services clearly increased during the following years (2022 and 2023). The percentage of infants visiting diagnostic audiological centers has improved over the years, from 20.73% during the first time interval (2020–2021) to 69.4% and 68.58% during the following time intervals (2021–2022 and 2022–2023, respectively).

## 4. Discussion

The aim of this study was to assess the performance of the Egyptian NHS program over a period of almost four years and to determine whether the program meets the JCIH guidelines’ recommended international benchmarks [10].

The major challenge for the ENHS is the extremely high birth rate, the short duration of hospital stays after delivery, and the numerous families living in remote areas who may have difficulties reaching a PHCU and even higher difficulties reaching an audiology department in a Ministry of Health hospital.

These factors played a major role in the selection of the screening method and the timing for the first screening. It was already known from conducting thyroid screening that it was not possible to perform the screening during the short hospital stay of one day after a normal delivery to a maximum of two days after delivery by Cesarean section. Therefore, and to ensure that the parents will come to the first hearing screening in a PHCU, hearing screening was combined with the established and mandatory thyroid screening that is performed for all infants at the age of one week.

The most important point for the success of any screening program is the presence of a good data management system that records data from all centers and tracks infants to minimize loss of documentation or loss to follow-up. A centralized screening center responsible for NHS was established at the Hearing and Speech Institute in Cairo and a computer data management system to connect all PHCUs with referral hospitals and the NHS center was developed by the Ministry of Health. The duties of the NHS center included supervising the screening units all over the country, receiving daily screening results, and tracking the neonates referred for a second screening. Furthermore, infants referred for diagnosis are tracked until a final diagnosis of the type and degree of hearing loss is reached and the results of the intervention are obtained.

### 4.1. Screening Method

The ENHS program started in 2019. During preliminary testing, both TEOAE and DPOAE were tested. TEOAE that is more sensitive than DPOAE in detecting mild and mild-to-moderate hearing loss showed a very high referral rate, mostly due to the noisy environment in PHCUs. This was the case despite assigning a separate, relatively quiet room for hearing screening. The DPOAE measurements were less vulnerable to ambient noise and showed much lower “REFER” rates. Considering the extremely high birth rate in Egypt and the fact that a high number of infants would fail the first screening using TEOAE (which the system may not be able to accommodate), it was decided to begin with implementing DPOAE testing. The screening goal was modified to detect infants with hearing thresholds of 50 dB or more. DPOAE testing has been implemented for NHS in some centers in countries with high birth rates such as in China and India [14,15]. Meanwhile, a smaller proportion of newborn hearing screening is conducted using DPOAE [16].

In the meantime, and to be able to detect all degrees of hearing impairment including mild hearing loss, efforts are made for better control of the testing environment. In several PHCUs, DPOAE testing is successfully replaced by TEOAE screening. Our aim is to implement TEOAE screening in all PHCUs all over the country. Feasibility studies are also being conducted with AABR screening in neonatal intensive- and intermediate care centers.

### 4.2. Coverage of Screening

Coverage of screening corresponds to coverage rate which is the number of infants screened divided by the total number of live births and should be ≥95% according to the JCIH guidelines.

The coverage rate of the ENHS was only 39% at the beginning of the program and increased over time to reach 82% in 2023. The percentage of infants who underwent screening varied between the different governorates. However, by the end of the evaluation period, the coverage rates of the different governorates were comparable. A low coverage rate at the start of the program (39%) was expected and has been reported in most programs. This is mostly attributed to a low acceptance of the parents and a low level of awareness of the importance of the screening in the beginning. System-related issues, such as inadequate screening environments (e.g., absence of quiet rooms), inexperienced screening staff, and inefficient institutional workflows, may also significantly influence screening outcomes [4]. One unexpected factor affecting the coverage rate was the COVID pandemic. The program was initiated in the first half of November 2019. In the almost 4 months until the beginning of March 2020, the number of newborns screened was 432.562 (64.3% of total births), while in the following 4 months, the number screened was only 240,330 (35.7% of total births).

Although there was a marked improvement in coverage rate by 2023 (82%), the minimum recommended benchmark of 95% has not been yet achieved.

In countries where UNHS has been implemented, the coverage rate varied. In parts of China, a coverage rate of 90.9% has been reported during a 5-year study from March 2002 until 2007 [17]. In Singapore, a UNHS was established in 2002. From April 2002 to March 2004, 99.8% of newborns were successfully screened [11]. In Malaysia and Iran, it took more than 10 years to reach the benchmark [13,18]. Neumann et al. [4] published a survey on the global status of newborn/infant hearing screening status of 158 countries from 2009 to 2019. Approximately one-third of the countries (accounting for 38% of the world population) had no/minimal screening and another third (33% of the world population) had near/fully implemented UNHS programs. Countries with fully implemented UNHS reported a coverage of screening ranging from 85% to 100%. Taking into consideration the extremely high birth rate in Egypt compared with most countries (around 2.5 million per year) and the short implementation time of the Egyptian program (since 2019), these results are very promising and indicate remarkable achievements for the program. The improvement in coverage rate over time indicates the adequate distribution of working centers throughout the country and that parents are beginning to accept the program. One crucial element for the parents’ acceptance is that the costs of the hearing screening and diagnosis are covered by the national health insurance. Friderichs et al. [19] demonstrated a lower coverage rate (22.5%) in clinics with non-permanent screening staff compared with clinics with dedicated personnel (74.6–84.7%). It is possible that the new personnel will require extra time to learn and adapt to the new responsibility. Since the beginning of the ENHS program, high-quality certification training of screening staff has been performed regularly and is one of the most important tasks of the screening tracking center. This could explain the continuous improvement in the results of the ENHS program.

### 4.3. Referral Rate After First Screening

The referral rate after first screening or initial referral rate is the percentage of newborns who failed the first screening in either one or both ears. According to the JCIH benchmark, it should be less than 4%.

The first screening in the Egyptian program is performed one week after delivery in PHCUs. The test is performed once. If noise or baby movement interrupts the test, it is repeated. A completed test with a “PASS” or “REFER” result is not repeated.

The initial referral rate varies considerably in other countries using OAE for screening such as South Africa (37.9%) [12], Uganda (43.3%) [20] and Brazil (30%) [21]. The OAE screening in these reports was usually completed in the maternity hospital within 12–24 h after birth due to early hospital discharge [21]. In the ENHS, the initial referral rate was 7.2% in 2019 and reached 5.2% in 2023, which is considered good. Van Dyak et al. [12] reported a high referral rate of 55% for healthy newborns in South Africa where infants were discharged from the hospitals between 6 and 24 h after birth. The high initial referral rate among newborns screened with OAE under 48 h after birth is attributed to the presence of vernix in the newborn’s external ear canal. Kumari and Rangasayee [22] reported that the vernix is still present in 35.4% of healthy newborns aged less than 24 h, causing transient conductive hearing loss and interfering with OAE screening and thus yielding false-positive results. Lupoli et al. [21] reported that delaying the screening test by 1 h resulted in a 5% decrease in the failure rate. One major discrepancy between the ENHS results and the above results is that in the ENHS program, the first screening is performed at the age of one week, thus leading to a lower initial referral rate. The results of the global survey published by Neuman et al. [4] found a mean fail rate of 4.5% (SD: 5.1; range: 0.2–30.8) for the newborn/infant hearing screening (NIHS) programs of all 55 reporting countries. The fail rate was statistically significantly lower in the countries with a high NIHS coverage of ≥85% (M = 3.1%, SD = 2.6, n = 33) and its range was narrower (0.3–11.6) compared with countries with a lower screening coverage (M = 6.5%; SD: 7.0; range: 0–98.2). The fail rate of the ENHS program improved over the years and reached 5.2% in 2023, which lies within the range reported for countries with high NIHS coverage in Neuman et al.’s study.

### 4.4. Follow-Up Rate for Second Screening

Follow-up rate is the number of infants that attended a second screening divided by the number of infants that failed the first screening (≥5%). Initially, NHS programs often have relatively high rate of infants who have failed the first screening and did not attend further testing (lost-to-follow-up rates). The common reasons for not coming to follow-up appointments include a long distance from the screening center, difficulties with transportation, fear and distrust in the governmental PHCUs and preferring to go directly to private physicians, a lack of awareness and misconceptions related to hearing loss, and inadequate visibility and availability of services [23].

The follow-up rate in the ENHS program improved throughout the study period, from 75.49 in the first year to 92.1% in the last year but it did not reach the benchmark of 95%.

A low percentage of infants attending follow-up would threaten the program’s effectiveness as it increases the number of children not being diagnosed for congenital hearing loss. At the beginning of the program, the loss-to-follow-up rate was high. However, a tracking system capable of minimizing documentation loss has been implemented and has helped in tracking these cases. An important aspect that needs to be considered is parental concerns. This is very important because false-positive results are inevitable. A false-positive result can cause unnecessary worries to the parents [8]. Some parents in our program were extremely worried, and they could not wait for the appointment for the second screening. Instead, they went directly to specialists in the private sector to have the hearing of their child evaluated. That is why the tracking team started contacting lost-to-follow-up cases all over the country. The tracking team found that some families completed a second screening in a private center and urged them to report the results to the PHCUs so the tests could be documented. Many families responded and reported the results of the second screening, which improved the percentage of children who attended follow-up.

Lost to follow-up is a problem reported in many countries besides Egypt. In a Malaysian study, the follow-up rate did not exceed 76.7%, resulting in an overall lost-to-follow-up rate of 24.2% [13]. Similar numbers were reported for UNHS programs in Italy [24], China [25], and Turkey [26]. Ahmed et al. [27] found that four factors contributed to poor follow-up rates: a lack of communication between the parents and screening personnel, ineffective protocols for scheduling follow-up appointments, parents’ lack of awareness regarding hearing loss and the need for early intervention, and transportation problems.

One strategy that can be used to improve this quality indicator is parental counseling during the first screening on the importance of the follow-up and the consequences of undetected or late detection of hearing loss in children [28,29].

Another solution is to align the second screening with routine immunization visits, which is carried out in the program in South Africa [30].

### 4.5. Rate of Referral for Diagnosis, Follow-Up Attendance Rate of Diagnostic Assessment and Lost-to-Follow-Up Rate for Diagnosis

According to the recommendations for the quality measures, the referral rate to diagnosis should range from 2 to 4%. During the evaluation period, the referral rate in the Egyptian program was less than 1.7%, which is lower than this benchmark. This may be due to the two-stage-screening protocol, which lowers the referral rate. It may also be linked to the high number of infants lost to follow-up after the initial screening [31]. Some parents worried by the failed first screening did not wait for the second screen and went directly to specialists outside of the Ministry of Health centers for diagnostic evaluation and did not report the results. We tried to solve this problem by having the tracking team contact the families and if they already had a diagnosis from the private sector, the results were input into our system. It is worth mentioning that the parents of children with confirmed hearing loss do report it to Ministry of Health hospitals in order to receive hearing aids and cochlear implants, which are fully covered by the national insurance. If they continue treatment in the private sector, they would have to pay for the treatment themselves.

The very low attendance rate in 2020–2021 (20.73%) and high lost-to-follow-up rate (79.27%) can be attributed to parents’ limited awareness of the importance of early follow-up and the consequences of delayed diagnosis, as well as the long distances to reach a specialized hospital. Only 12 equipped Ministry of Health hospitals were available for diagnostic evaluation from 2020 to 2021. Moreover, at the beginning of the program, prior to using computerized documentation, the documentation was performed manually. This may have led to incomplete documentation of diagnostic results.

At the same time (2020–2021), during the COVID pandemic, many hospitals became isolation centers and diagnostic audiological testing was not possible.

The number of infants visiting the hospital and receiving diagnostic services increased during the following years (2022 and 2023). The percentage of infants who visited diagnostic audiological centers was 20.73% (out of the infants referred for diagnosis) during the ENHS program’s first year (2020–2021). This improved remarkably during the following years, reaching 68.58% during the third to fourth year (2022–2023). However, this number is lower than the 90% JCIH benchmark or even the more feasible benchmark of 70% suggested by Ravi et al. [29]. This problem exists in other countries as well. According to the survey on the global status of hearing screening, Neumann et al. [4] reported that in countries with near/fully functioning UNHS programs, the lost-to-follow-up rate was 7% on average and was lower than in countries with a lower UNHS coverage. Of the 27 countries that reported trustworthy lost-to-follow-up rates, 13 (48%) had rates above 30%. Bussé et al.’s [32] meta-analysis found lost-to-follow-up rates above 30% in nearly half of the studied NIHS programs. This indicates that many children with suspected hearing loss are still undiagnosed. The reasons for this high lost-to-follow-up rate are similar to those mentioned in the previous section including a lack of communication between the parents and screening personnel, ineffective protocols for scheduling follow-up appointments, a lack of awareness of the parents regarding hearing loss and the need for early intervention, and transportation problems [27].

The most commonly used or suggested measures to increase follow-up include a dedicated multidisciplinary team, a focus on public awareness, and improving documentation and database systems [29].

### 4.6. Intervention Policies for Childhood Hearing Loss in Egypt

Infants diagnosed with permanent hearing loss should receive an intervention as soon as possible, which includes receiving hearing aids and/or medical treatment for middle ear ventilation disorders. Cochlear implants are performed in children diagnosed with severe-to-profound hearing loss who did not benefit from hearing aids following a trial period. All treatment modalities, including medical treatment, hearing aids, and cochlear implants, are fully covered by the national insurance in Egypt. The effective coverage of newborn hearing screening services within the population (i.e., the proportion of infants with permanent childhood hearing loss who received appropriate interventions within the first six months of life according to Neumann et al. [4] was not investigated in this work. This topic is being investigated by a separate group from the Egyptian Ministry of Health.

## 5. Conclusions and Recommendations

The Egyptian UNHS program only started recently. Within a short time, the program was able to serve more than five and half million live births. This study shows that during the initial phase, the results did not reach the recommended JCIH benchmarks (percentage coverage, initial referral rate, and follow-up rate) but there was continuous improvement in the quality measures. The number of newborns who did not attend the follow-up screening (lost-to-follow-up), which could affect the program’s effectiveness regarding early diagnosis and intervention, markedly decreased. This was possible through the application and continuous improvement of the tracking system developed by the Ministry of Health.

Parental understanding and cooperation are very important for the success of NHS. Public awareness programs, for example, through social media, about the consequences of hearing loss and the importance of early intervention are of utmost importance and are being developed. Specialists from the Hearing and Speech Institute, audiology departments in different universities, and the Egyptian Audio-vestibular Medicine Association (EAVMA) give numerous interviews on TV and radio channels. Celebrations are performed on occasions such as World Hearing Day with round table discussions including representatives from the medical field and social sector, politicians, and parents improve public awareness. Clergymen from religions practiced in Egypt are specifically invited and encouraged to attend such occasions since they have access to and a great influence on the public, especially in rural areas.

Shortening appointment times and providing quick and easy access to diagnostic audiology centers is essential for rapid and accurate diagnoses. Expansion of diagnostic and intervention centers and training of specialists in pediatric audiological diagnosis are also recommended.

## Figures and Tables

**Table 1:** Number and percentage of babies who failed the first screening and were referred for a second screening.

	Total Number of Babies Screened	Number That Failed First Screening	Referral Rate After First Screening
2019–2020	749,872	54,510	7.27%
2020–2021	1,061,268	53,075	5.00%
2021–2022	1,518,801	83,303	5.48%
2022–2023	1,846,114	94,526	5.12%
Total	5,176,055	285,414	5.51%

**Table 2:** Number of neonates referred to and attended a 2nd screening, follow–up rate, and lost-to-follow-up rate for 2nd screening.

Time Interval	Number Referred for Second Screening	Number That Attended Second Screening	Follow-Up Rate	Lost-to-Follow-Up Rate
2019–2020	54,510	41,151	75.49%	24.51%
2020–2021	53,075	40,527	76.35%	23.65%
2021–2022	83,303	65,729	80.55%	19.45%
2022–2023	94,526	87,061	92.10%	7.90%
Total	285,414	234,423	82.13%	17.87%

## Data Availability

The datasets presented in this article are not readily available due to privacy restrictions. Requests to access the datasets should be directed to the Egyptian Ministry of Health, Cairo, Egypt.
